# Video consultation in the prehospital stroke chain of care for suspected large vessel occlusion: a mixed-methods feasibility study

**DOI:** 10.1186/s12873-026-01575-y

**Published:** 2026-04-11

**Authors:** Lise-Lotte Omran, Hanna Maurin Söderholm, Magnus Andersson Hagiwara, Bengt Arne Sjöqvist, Annika Nordanstig, Goran Puaca

**Affiliations:** 1 PreHospen Center for Prehospital Research, University of Borås, Borås, 501 90 Sweden; 2 Lindholmen Science Park, PICTA Prehospital Innovation Arena, Göteborg, Sweden; 3 Department of Electrical Engineering, Chalmers University of Technology, Gothenburg, Sweden; 4 Department of Neurology, Sahlgrenska University Hospital, Gothenburg, Sweden; 5 Department of Clinical Neuroscience, Institute of Neuroscience and Physiology, Sahlgrenska Academy at University of Gothenburg, Gothenburg, Sweden; 6 Faculty of Caring Science, Work Life and Social Welfare-Department of Work Life and Social Welfare, University of Borås, Borås, Sweden

**Keywords:** Ambulance nurse, Emergency medical services (EMS), Feasibility study, Interprofessional collaboration, Prehospital care, Stroke, Telemedicine, Video consultation

## Abstract

**Background:**

Early recognition and decision-making are critical in acute stroke care. Prehospital video consultation may support collaboration and inform triage decisions between ambulance nurses (ANs) and neurologists. However, organizational and geographical factors may delay access to reperfusion therapies. This feasibility study explored whether prehospital video consultation between ANs and neurologists could be implemented in routine stroke care and how such consultations might influence workflow and perceptions of patient safety.

**Methods:**

We conducted a mixed-methods feasibility study with parallel collection of quantitative and qualitative data. Quantitative data on processing times and patient flow were extracted from ambulance and hospital records. Qualitative data were obtained from semi-structured interviews and evaluation forms. The intervention was implemented in 12 ambulances across four districts over a 13-month period. Patients with suspected large vessel occlusion (LVO) and a modified NIH Stroke Scale (mNIHSS) score ≥ 6 were eligible.

**Results:**

Forty-four patients met the inclusion criteria; however, only five video consultations were successfully completed, partly due to limited equipment availability. Quantitative findings were therefore presented descriptively. Among the five completed video consultations, two patients were transported directly to the regional stroke center, compared to 24 of 148 patients in the non-video group. The qualitative analysis identified three themes: (1) interprofessional collaboration and trust (2), practical use of video, and (3) barriers and facilitators to implementation.

**Conclusions:**

Prehospital video consultation in suspected stroke was technically feasible in individual cases and perceived as supportive by clinicians. However, technical and organizational barriers substantially limit routine use. The findings provide important insights into professional, organizational, and technical factors influencing feasibility, but do not permit conclusions regarding clinical effectiveness.

**Clinical trial number:**

Not applicable

## Background

Stroke remains a leading cause of death and long-term disability worldwide and imposes a substantial societal and economic burden [1]. In the acute phase, patient outcome is highly dependent on rapid access to appropriate treatment. The vast majority of strokes are ischemic in origin, and current therapeutic strategies include intravenous thrombolysis and endovascular thrombectomy [2]. While thrombolysis is widely available at emergency hospitals in Sweden, thrombectomy is performed only at a small number of specialized centers [3]. Outcomes are particularly time-dependent for patients with large vessel occlusion (LVO), which places high demands on early recognition and accurate prehospital triage to ensure transport to the most appropriate facility without unnecessary delay. As a result, the organization of the stroke care pathway particularly during the early, prehospital phase plays a crucial role in determining whether patients receive timely and effective treatment. Accurate prehospital identification of stroke severity and possible LVO is therefore essential for appropriate triage and destination decisions [4]. For patients eligible for thrombectomy, the therapeutic benefit diminishes rapidly as time passes, meaning that even relatively short delays may have significant clinical consequences [5]. From a broader perspective, prolonged time to treatment also leads to increased societal costs through extended rehabilitation needs and long-term dependency [6].

The prehospital phase represents the first point of medical contact for most stroke patients and is therefore a critical window for intervention [7]. Decisions made by emergency medical services (EMS) personnel regarding assessment, prioritization, and hospital destination can strongly influence the subsequent course of care [4]. Ideally, patients with a high likelihood of large vessel occlusion (LVO) should be identified early and transported directly to a hospital capable of performing thrombectomy [2]. Such an approach aims to avoid unnecessary intermediate stops at hospitals without thrombectomy facilities, which often result in substantial delays due to initial evaluation, imaging, and coordination of secondary transport. Interhospital transfers following primary admission to a non-thrombectomy hospital are associated with considerable time loss [8, 9]. Given the steep time-outcome relationship in thrombectomy, these delays can reduce the likelihood of favourable outcomes and, in some cases, exclude patients from treatment altogether. Current national guidelines recommend initiation of thrombolysis within 4.5 h of symptom onset and thrombectomy within 6 h, with extended time windows applicable to selected patients based on advanced imaging criteria [10].

Despite the clear advantages of early identification and direct transport, accurate prehospital triage for thrombectomy remains challenging. In contrast to acute myocardial infarction, where prehospital electrocardiography is a central diagnostic tool [11], stroke diagnosis relies primarily on clinical assessment. However, prehospital identification of myocardial infarction is also influenced by clinical symptoms, patient history, timing of symptom onset, hemodynamic status, and interpretation of ECG findings, which may be subject to variability and uncertainty. Thus, both conditions require complex clinical judgment in the prehospital setting. In stroke care, prehospital stroke scales and telephone consultations with stroke specialists are commonly used, but these methods are subject to uncertainty and variation in experience, increasing the risk of both over- and undertriage [12]. In Sweden as in most of developed countries, EMS has evolved into a highly specialized field. Ambulances in Sweden are staffed by registered nurses, often with additional postgraduate education in prehospital emergency care, qualifying them as ambulance nurses (ANs). The second crew member is either another AN or an emergency medical technician. In cases involving severely ill patients, ANs carry medical responsibility and conduct assessments and interventions in accordance with national and regional clinical guidelines [13]. Over time, their role has expanded beyond transport to include advanced clinical decision-making, including selection of care pathways and hospital destinations based on the patient’s condition.

When stroke is suspected, established protocols guide prehospital management. ANs are required to consult a physician typically a stroke neurologist in the nearest local hospital before activating the stroke pathway. This consultation is currently performed by telephone. Patients are generally transported to the nearest hospital offering thrombolysis, where brain imaging is performed to confirm diagnosis and assess treatment eligibility [3]. If imaging reveals an LVO and the patient meets criteria for thrombectomy, a secondary transfer to a regional stroke center is initiated. Although this pathway ensures access to both treatments, it often leads to avoidable delays for patients ultimately requiring thrombectomy.

Research has shown that EMS personnel are generally proficient in recognizing stroke symptoms, and early recognition is strongly associated with faster access to diagnostic imaging and treatment [7]. Conversely, missed or delayed identification of stroke in the prehospital phase is linked to longer treatment times and increased mortality [4]. Given that approximately three quarters of stroke patients are first assessed by EMS, even modest improvements in prehospital processes have the potential to produce meaningful clinical benefits on a population level.

Several strategies have been proposed to address delays in the stroke care pathway. One approach is the use of Mobile Stroke Units (MSUs), which are equipped with on-board CT scanners, laboratory facilities, and specialized staff. MSUs have been shown to reduce time to thrombolysis and may facilitate earlier thrombectomy decisions [14]. However, their high costs and logistical demands limit feasibility, particularly outside densely populated urban areas. Previous feasibility studies of prehospital video consultation have primarily focused on technical aspects. Video consultation has been proposed as a potential tool to support prehospital neurological assessment and triage in suspected stroke [15–17].

Introducing video consultation into the prehospital stroke care pathway represents a complex intervention. Evidence regarding routine implementation remains limited, warranting smaller-scale feasibility evaluations.The purpose of this study is therefore twofold: the primary aim of this feasibility study was to explore whether real-time video consultation between ANs and neurologists could be conducted during prehospital assessment of patients with suspected LVO. A secondary, exploratory aim was to examine how video consultation may affect interprofessional collaboration, workflow, and clinical decision-making.

## Method

### Study design

This was a mixed-methods feasibility study using a non-randomized, quasi-experimental approach. The study was guided by the GRAMMS reporting guideline for mixed methods studies [18]. The study followed a convergent parallel mixed methods design, in which quantitative and qualitative data were collected concurrently, analyzed separately, and integrated during interpretation, consistent with established mixed methods frameworks [19]. The study was conducted over a 13-month period (December 2021 – December 2022).

### Participants and settings

The study was conducted in Västra Götalandsregionen (VGR), one of Sweden’s largest healthcare regions, covering both urban and rural areas in western Sweden with approximately 1.7 million residents. The region includes seven local hospitals with thrombolysis capacity and one regional stroke center at Sahlgrenska University Hospital in Gothenburg, which performs all thrombectomies. Ambulance services in VGR are hospital-based and organized under four main districts corresponding to the local hospitals. This organizational structure, combining local autonomy with access to regional specialist expertise, made the region suitable for testing the feasibility of prehospital video consultation in suspected stroke. The intervention was implemented in twelve ambulances across four districts belonging to the same regional stroke center.

In suspected stroke cases, regional guidelines require ANs to conduct an initial structured assessment using the Airway, Breathing, Circulation, Disability, and Exposure (ABCDE), framework followed by evaluation using the modified National Institutes of Health Stroke Scale (mNIHSS) The mNIHSS is a simplified version of the NIHSS used to assess stroke severity. It includes fewer items, focusing on the most clinically relevant neurological deficits, which makes it faster to perform and improves inter-rater reliability.

In this study, the mNIHSS was based on previously validated versions of the scale [20, 21] but applied in accordance with the regional “Rädda hjärnan” (Save the brain) protocol in the region of Västra Götaland. According to this protocol, the mNIHSS included assessment of: (1) orientation (current month and patient age) (2), simple commands (“close your eyes” and “make a fist”) (3), gaze (4), visual fields using a finger-wiggling test (5), motor function of the arms (6), motor function of the legs (7), sensory function in the hand and foot, and (8) language/aphasia. Compared with the full NIHSS, the mNIHSS excluded formal assessment of facial palsy, dysarthria, extinction/inattention (neglect), and detailed level of consciousness scoring [22]. This modified scale was chosen since it already is a part of the guidelines regarding the assessment of suspected stroke in the region.

If the mNIHSS score is ≥ 2 points and the estimated transport time to the regional stroke center exceeds 45 min, the guidelines state that the AN must contact the nearest hospital for initial evaluation and computed tomography (CT) imaging. If the imaging confirms a LVO, and the patient is considered eligible for thrombectomy, a secondary ambulance transfer to the regional stroke center is initiated [3, 23].

Under standard care, prehospital neurological assessment relied on the mNIHSS performed by the ambulance nurse and telephone consultation with a neurologist. In the video intervention, this process was expanded through real-time video consultation, which enabled the on-call neurologist to directly observe the patient and guide the ambulance nurse through a full NIHSS examination in addition to the initial mNIHSS. Thus, the key difference between standard care and the intervention was that the video group allowed remote specialist-led assessment using the complete NIHSS, whereas the control group was limited to mNIHSS and telephone-based consultation only. Based on this real-time video assessment and the full NIHSS, the neurologist on call could decide on direct transport to the regional stroke center when deemed appropriate, even in situations where transport time would otherwise have led to initial evaluation at a local stroke center.

The design of the video consultation system was informed by a preceding simulation phase within the same research project, in which prehospital video support for stroke consultation was evaluated and the value of multi-angle video streams for remote neurological assessment and collaborative decision-making was identified. The intervention ambulances were equipped with three fixed cameras providing different views, one providing a close-up of the patient’s face, one side view for observing limb movement, and one wide-angle lens showing the entire ambulance environment, enabling real-time neurological assessment. Neurologists accessed the system through a secure tablet. The video system was configured to enable assessment of key neurological functions, including facial expression, eye movements, speech, and limb motor function during the NIHSS examination. Both neurologists and ANs received short training sessions and were instructed to revert to telephone consultation if technical issues arose [24]. Video Support in the PreHospital Stroke chain (ViPHS) has been developed as a pragmatic video consultation system designed for use in standard ambulances [25]. The system enables real-time video communication between the ambulance crew and an on-call stroke neurologist, allowing the neurologist to remotely perform a structured neurological examination using the NIHSS [26]. The intended purpose of ViPHS aims to improve the identification of patients likely to benefit from thrombectomy and support informed decisions regarding direct transport to a thrombectomy-capable center. In the prehospital stroke context, video consultation introduces a new mode of interprofessional collaboration between ANs and neurologists.


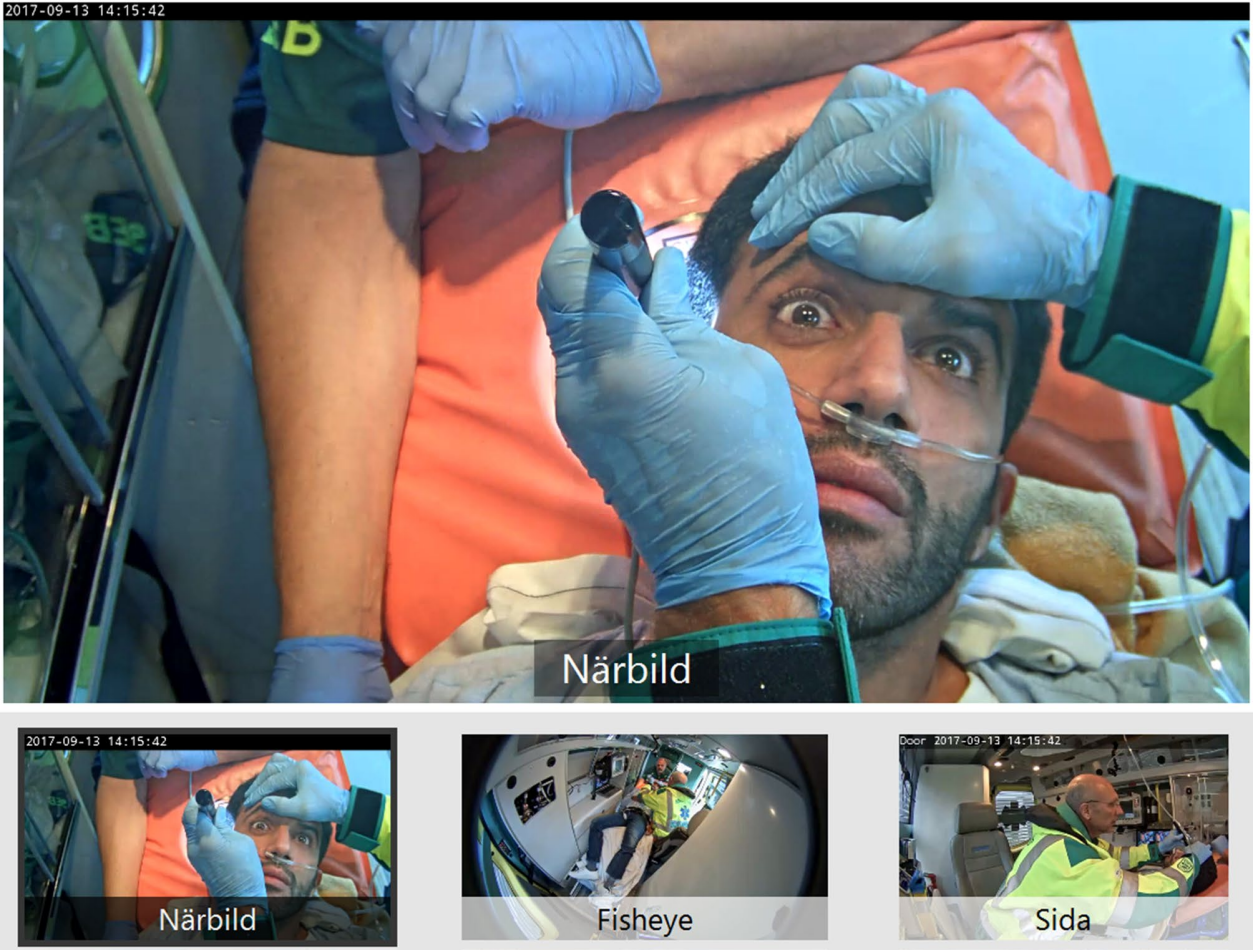


The images originate from a simulation study conducted in 2017 and illustrate the technical setup and camera perspectives of the video consultation system. The system configuration remained unchanged during the study period (2021–2022), making the images representative of the intervention used in the present study. ”Swedish terms in the figure indicate camera perspectives with closeup (närbild) Fisheye and side view (sida).

The video consultation system (ViPHS) used a secure, encrypted connection between the ambulance and the on-call neurologist at the regional stroke center. The platform was hosted on a secure regional healthcare server managed by Västra Götalandsregionen, in accordance with regional data protection and patient safety standards. Video transmission relied on routinely available mobile network connectivity in the region, allowing stable real-time data transfer between the ambulance and the neurologist. During the consultation the neurologists could switch between camera views and guide the AN through a structured neurological examination, including a full NIHSS assessment. The NIHSS is a comprehensive neurological scale assessing level of consciousness, language, motor and sensory function, visual fields, and neglect [27], and was used to support triage decisions and determine the most appropriate destination hospital. Audio communication occurred via the existing ambulance communication network, allowing both parties to speak freely while maintaining stable image transmission.

According to the regional ViPHS guidelines, a video consultation was initiated for patients with suspected LVO and a mNIHSS score of ≥ 6. The neurologist advised on triage and destination direct transfer to the thrombectomy center or the nearest thrombolysis-capable hospital. In the event of technical failure, the team attempted one reconnection before reverting to standard telephone consultation, ensuring patient safety and workflow continuity.

### Inclusion criteria and sampling

Patients were eligible for inclusion if they met the following criteria:

1. Documented stroke symptom onset within the last 4 h;
2. A mNIHSS score of six points or higher; and
3. Preserved level of consciousness.

Altogether, 152 patients with suspected stroke were screened during the study period. Patients were excluded if they presented decreased consciousness, deteriorating vital signs, logistical or technical barriers preventing video consultation.

The intervention group consisted of patients who fulfilled the inclusion criteria and were transported by ambulances equipped with video and where a video consultation was conducted. Whereas the control group comprised patients who met the same inclusion criteria but were transported by ambulances following standard telephone consultation.

The quantitative component, allocation to the intervention or control group was determined by routine ambulance dispatch procedures rather than randomization. Patients were assigned to the video consultation group if the nearest available ambulance was equipped with the video consultation system, while patients transported by ambulances without video equipment constituted the control group. This allocation reflects real-world operational conditions but may introduce selection bias related to geography or ambulance availability.

### Data collection

The quantitative data for this study were collected from ambulance and hospital records by an individual familiar with the documentation systems, following a predefined list of variables. These variables included, for example, time of ambulance dispatch, arrival at the patient, departure from the scene with the patient, the ambulance nurse’s initial mNIHSS assessment, and the receiving physician’s first NIHSS assessment at the hospital. Most variables were documented in the ambulance records; however, mNIHSS scores were in some cases recorded as free text rather than using a standardized protocol. The receiving hospital’s NIHSS assessment was likewise extracted from free-text documentation in the hospital medical records. The qualitative component was based on individual semi-structured interviews conducted both via video and on site.

The interviews were conducted shortly after video consultations had taken place and included both the AN and the neurologist involved in each case. All personnel who had participated in the consultation were asked whether they were willing to take part in the study. They were contacted via email and received written information about the study, including that their participation was voluntary and that they could withdraw at any time. This information was also provided orally prior to the start of each interview. The interviews explored perceptions of collaboration, workflow, decision-making, and the practical use of video consultation in prehospital stroke care. In total, two ambulance nurses and five neurologists (one interview represented two consultations) participated in the interviews. Other eligible ambulance nurses declined participation. The interviews lasted approximately 45–60 min each.

ANs and neurologists were also asked to complete an evaluation form after a video consultation was initiated. The evaluation form consisted primarily of multiple-choice questions assessing aspects such as video quality, interprofessional collaboration, and technical performance, with response options ranging from *poor*, *less good*, *acceptable*, *very good*, to *excellent*. The form concluded with an open-ended question allowing participants to provide free text comments. The combination of interviews and evaluation forms allowed for triangulation of qualitative and quantitative insights.

Of the 152 patients meeting the inclusion criteria, 44 were transported in video-equipped ambulances. Five video consultations were included in the quantitative data, while one additional consultation was excluded due to a lower mNIHSS score, though an interview was still conducted since a consultation had occurred. In total, 15 evaluation forms from ambulance nurses and nine from neurologists were completed, representing 20 consultation attempts, including both successful and unsuccessful attempts performed.

**Fig. 1:**
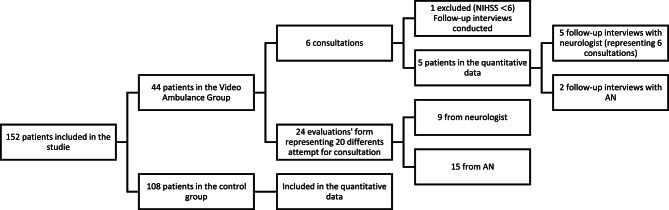
Flowchart of included patients

### Data analysis

Due to the limited sample size and non-normal distribution of the data, continuous variables were summarized using median values and ranges, while categorical variables were reported as frequencies (N) and percentages (%). Due to the small sample size, analyses were descriptive only, no statistical tests were applied. All statistical analyses were performed using IBM SPSS Statistics, version 28.

Interview data were analyzed using inductive thematic analysis following Braun and Clarke’s six-phase approach (2006) [28]. The audio-recorded interviews were transcribed verbatim and analyzed through coding and theme development. One researcher conducted the initial coding and analysis, coding the transcripts to generate initial codes. These codes were iteratively grouped into analytic categories and developed into candidate themes. Throughout the analytic process, emerging codes, categories, and themes were discussed within the multidisciplinary research team. These discussions were used to challenge interpretations, refine theme boundaries, and enhance credibility and interpretive consistency. This collaborative reflection supported analytical rigor while maintaining a coherent analytic perspective.

Evaluation forms were analyzed using a mixed approach. Closed-ended questions were analyzed descriptively to summarize quantitative aspects such as reasons for unsuccessful consultations and ratings of video quality, collaboration, and technical performance. Responses to the open-ended question were analyzed qualitatively using the same inductive thematic approach as the interview data and were used to complement and triangulate the interview findings.

### Ethical considerations

The study was approved by the Regional Ethics Review Board in Gothenburg, Sweden (Ref. No: 2018-06-18). Written and verbal consent was obtained from all participants involved in the qualitative interviews (ANs and neurologists). For patients included in the quantitative component, informed consent was waived by the Regional Ethics Review Board due to the observational design and the use of pseudonymized data. The images in the manuscript are derived from simulation scenarios and do not show real patients. The individuals are neurologist acting as patients. Written informed consent for publication of illustrative images was obtained from all individuals appearing in Fig. 1. No patient-identifiable data are included in this manuscript.

All standard procedures were followed throughout the study period, with the only modification being that clinical consultations were conducted via video instead of telephone, which remains the standard practice. ANs received instructions on how to use the video consultation system. They were advised to follow their normal assessment routines, with the exception that if a patient scored six points or more on the mNIHSS scale, they were to abort the on-scene examination, transfer the patient to the ambulance, and initiate the video consultation from inside the vehicle. In the event of technical issues, they were instructed to attempt reconnection once. If the video connection could not be re-established, they were to revert to standard telephone consultation.

The study protocol included a provision for early termination if substantial delays were detected. No video material was recorded or stored. All data were pseudo-anonymized, and identifying information was kept in a separate code key stored securely and separately from the main dataset. The study was conducted in accordance with the ethical principles outlined in the Declaration of Helsinki [29].

## Results

### Patient management

Both the control group and the video consultation group were comparable in terms of age, gender, mNIHSS and initial NIHSS scores. The total median prehospital time was 83 min (43–86) in the video consultation group and 56 min (17–132) in the non-video group. The median on-scene time was 24 min [16–40] in the video consultation group and 23 min (5–55) in the non-video group (see Table 1).

**Table 1:** Prehospital time and time to treatment

Variable (Minute) median (min-max)	Video consultation (*N* = 5)	Non video consultation (*N* = 148)	Missing data
Time to patient	12 (10–18)	13 (2–43)	0
Time on scene	24 (16–40)	23 (5–55)	12
Time to hospital	40 (14–50)	19 (1–64)	12
Total prehospital time	83 (43–86)	56 (17–132)	10
Time from ambulance assignment to CT-brain (*N* = 153)	95 (54–131)	71 (28–223)	2
Time from ambulance assignment to thrombolysis (*N* = 44)	102 (102)	76 (31–181)	
Time from ambulance assignment to thrombectomy (*N* = 48)	182 (138–226)	169 (98–283)	

*=Difference between median

Among the five patients in the video consultation group, two were transported directly to the regional stroke center, compared with 24 of 148 patients in the non-video group. Given the very small sample size, these proportions are presented descriptively in Table 2. Of the two patients transported directly to the regional stroke center, one did not receive thrombolysis because imaging revealed a hemorrhagic stroke.

**Table 2:** Patient destination and received treatment

Variable *N* (%)	Video consultation (*N* = 5)	Non-video consultation (*N* = 148)	Missing data
Local hospital (*N* = 127)	3 (60)	124 (83)	0
Regional stroke center direct (*N* = 26)	2 (40)	24 (16)	0
Regional stroke center secondary (*N* = 38)	1 (21)	37 (25)	0
Thrombolysis (*N* = 47)	1 (20)	46 (31)	0
Thrombectomy (*N* = 49)	2 (40)	47 (32)	0
Thrombectomy direct (*N* = 12)	1 (20)	11 (7)	0

Given the very small number of completed video consultations (*n* = 5), these cases should primarily be interpreted as illustrative rather than as a basis for statistical inference.

### Result from evaluation forms and screening

**Fig. 2:**
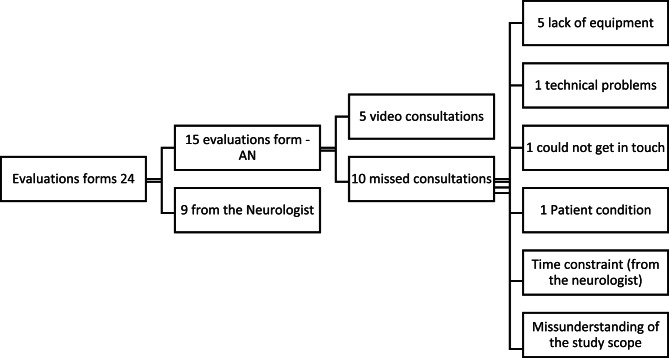
Summary of evaluation forms

Of the 44 patients included in the study, 24 evaluation forms were available for 20 cases. According to the evaluation forms, the most common reason for a missed consultation was that the on-call neurologist did not have access to the tablet, accounting for 50% of the missed consultations among those with available feedback, see figure 2. Other reasons reported in the evaluation forms included one case with technical problems where the tablet failed to connect with the ambulance, one case in which the ambulance nurse reached the neurologist but no consultation was conducted due to workload, and one case where the neurologist believed the study was limited to office hours. Figure 2 summarizes reasons for failed or incomplete video consultations based on evaluation forms. During the implementation period, ambulance records were systematically reviewed to identify potential patient safety risks. The review revealed that ambulance nurses attempted to initiate video consultations in nearly all patients who met the inclusion criteria; however, many of these attempts were unsuccessful due to a range of factors related to the neurologist on call.

### Ambulance nurses’ and neurologists’ perspective on video consultation – results from the interviews

The results are presented in three themes: (4.3.1) Interprofessional collaboration and trust, (4.3.2) Collaboration and use of video, and (4.3.3) Barriers and facilitators in the ViPHS project. The first theme describes mutual trust, clear role distribution, and differences in adherence to routines. The second highlights that video consultations offered equal interaction to traditional calls, with added visual benefits but also practical and technical challenges. The third outlines factors that supported or hindered implementation, including staff engagement, patient benefits, limited awareness, low frequency of use, and technical issues.

#### Interprofessional collaboration and trust

Neurologists emphasized the importance of a well-functioning collaboration and highlighted that ambulance services constitute a central part of the stroke care chain. They expressed strong trust in the assessments made by ANs, stating that these evaluations are usually accurate. Several neurologists speculated that stroke assessments might sometimes be less reliable when performed by other, less experienced physicians.

*…the ambulance staff are probably more consistent*,* more uniform. Most of them are relatively experienced*,* they know quite well what information is needed*,* and they provide good answers. […] It can actually be more difficult with colleagues [laughs]*,* because there is more variation […] for example*,* a resident who works in gastroenterology and has very limited experience of stroke. (N2)*

Clear role distributions were seen as a prerequisite for effective collaboration. Although ambulance personnel sometimes questioned certain routines, both those and physicians’ decisions were respected. The neurologists noted that it could be more challenging to collaborate with, for example, helicopter doctors, who occasionally deviated from routines without first consulting the receiving physician.

*The nurse is more likely to accept that we have these guidelines or care pathways and will follow it for the most part. In contrast it is more common for doctors to just make up their own (laughs) ways of doing things. (N6)*

From the ANs perspective, the neurologist´s attitude during consultation was described as both respectful and attentive, and they felt that their clinical judgment was valued.

*// she listened to what I said and did not make any judgement … she respected what I said and did not question my assessments or how we had arrived at the decisions we made. I think that shows that you respect your counterpart*,* or your colleague*,* or whatever you want to call it. (AN 1)*

ANs described having limited influence over the development of routines and guidelines. It emerged that incident reporting is often their only tool for trying to impact decisions, but it can be difficult to have their voices heard higher up in the organization. They also found it disruptive when different hospitals applied different guidelines for similar situations.

#### Collaboration and use of video

Both neurologists and ANs agreed that their collaboration during video consultations worked just as well as during traditional consultations, with interaction described as equal and respectful. ANs helped to manage the camera and facilitated the video assessment, when the neurologist couldn’t manage the controls.

*I actually wasn’t able to operate the camera at all; they did that on site. The ambulance staff helped me with it. (N1)*

Video consultation was perceived as particularly beneficial when symptoms were difficult to describe verbally, as visual information complemented verbal reports and reduced the risk of misunderstandings. Seeing the same thing in real time enabled joint assessments and strengthened mutual understanding of both the patient’s condition and the prehospital context

*Then you don’t have to explain as much … ‘It kind of connects*,* I think*,* maybe with …’ Yes*,* then they can see it themselves. So yes*,* I think that’s it … that it’s good. (A5)*

Neurologists found it easier to guide ANs through specific examinations using video, and the shared visual reference improved mutual understanding of the patient’s condition and the prehospital context. This was especially valuable in uncertain cases, enabling more confident joint decision-making.

*I don’t think it’s so much about me just seeing and assessing*,* because I think we’re looking together in a different way and can make the assessment together. (N4-5)*

One neurologist also described that seeing the patient created a stronger memory of the case and a more complete impression than telephone consultation alone.

*I will remember her better [laughs] simply because I got to see her as well. (N6)*

At the same time, both groups noted that video technology could occasionally distract the patient and the medical content in the ambulance environment. ANs described that video sometimes interrupted their usual care flow, especially in home settings, and could require them to take on a more active role in leading the consultation, which was demanding without clear leadership.

*Now we’re going to… Now we’re reaching these points. Now we have to wrap up and get to the ambulance and start the video assessment. And then you lose a bit of treatment pace*,* and you lose a bit of initial decisiveness… // It became… a bit confusing… You don’t finish your own assessment… // It’s that there’s such a sudden shift in tempo… before you get into it… and feel that you’re back in your own local routines. (A1)*

Technical usability issues were also reported. Problems such as switching views or zooming could delay consultations, and the tablet device was considered impractical to carry during hospital rounds, leading to it often being left in the office.

*…it’s a bit of a hassle to carry around this tablet [laughter]… because right now it’s quite heavy and bulky. // And that can have some impact*,* because you forget to bring it*,* since you’re often doing other things even when you’re the on-call consultant. // And it’s the same during the day when you’re the acute stroke doctor - it’s a bit bulky to carry it around.(N3)*.

#### Barriers and facilitators in the ViPHS

Participants acknowledged both practical challenges and clear benefits of the ViPHS project. On the positive side, ANs felt engaged in the initiative and believed they could influence its implementation. They valued the potential of video consultation to benefit patients particularly by avoiding unnecessary intermediate stops in the care chain. Both groups maintained that it remained important for patient care.

*I can imagine that it also sends signals to those working in the ambulance that stroke*,* acute stroke management*,* is important*,* // It puts the focus on stroke in some way*,* and that’s only a good thing*,* I think. (N6)*

Barriers included inconsistent awareness of the project among other hospital staff, such as primary on-call neurologists and stroke unit nurses, which sometimes caused delays or repeated examinations upon hospital arrival. Neurologists also reported that video consultations were relatively infrequent, making it difficult to maintain proficiency, and requested more training opportunities. Simplified inclusion criteria were suggested to improve clarity and uptake.

*It didn’t turn out ideally*,* because I think that if we had arrived and it had been the neurologist who made the decision after the video consultation*,* she could have started the scan right away. Instead*,* it was more like*,* ‘Oh*,* okay.’ Then a different neurologist still wanted to take a quick look at the patient*,* which made things somewhat unclear. Also*,* the rest of the stroke unit staff did not know on what grounds we had arrived*,* so in the end the video assessment felt a bit strange. A1*.

A significant challenge in facilitating consultations was the practical implementation of the solution. The technical system was perceived as difficult to use, and the tablet was regarded as impractical to carry both when on call outside the hospital and during primary duty within the hospital.

*Being in the ER*,* making a call and … then running into a room and … I think that could actually be a bit stressful. But then it’s the stroke doctors who might have it at home. I’ve also noticed that … most of them thought it was such a small hassle that they probably didn’t take it home. N2*.

Hierarchy in clinical practice was generally not perceived as a barrier in video consultations. Participants described clear roles and a shared goal of serving the patient’s best interest. Neurologists acknowledged that hierarchies exist in healthcare and can occasionally hinder communication, particularly when roles are unclear, but in acute situations such structures were viewed as necessary because the physician bears ultimate medical responsibility. With greater experience, both neurologists and ANs felt it became easier to look beyond hierarchical boundaries in collaborative decision-making.

*She respected what I said and listened and did not question my assessments or how we had arrived at the decision that it would be as it turned out. I think that then you will respect your counterpart*,* or your partner*,* or whatever you want to call it. (A1)*

## Discussion

This mixed-methods feasibility study explored real-time video consultation between ANs and neurologists in suspected stroke. While the intervention was technically feasible and perceived as clinically useful, the very small number of completed video consultations limits conclusions regarding effectiveness or routine feasibility. Rather than demonstrating readiness for large-scale implementation, the findings primarily provide insight into professional, organizational, and technical factors influencing the use of prehospital video consultation in real-world stroke care [30–32]. The study should therefore be interpreted as exploratory. Accordingly, the five video consultations should be understood as illustrative cases reflecting real-world operational conditions.

The longer median prehospital and process times observed in some of the video consultation cases should be interpreted with caution. Given that only five video consultations were completed, these differences likely reflect logistical variation between districts rather than a true systematic delay caused by the intervention itself. Importantly, the median on-scene time was similar between the video and non-video groups, suggesting that the video consultation itself did not substantially prolong time spent with the patient at the scene. Previous simulation-based studies of prehospital video consultation have indicated that the use of real-time video consultation may introduce additional steps related to assessment and coordination [24], which may influence on-scene time. In the present study, on-scene time remained comparable between groups, suggesting that video consultation can be integrated without substantial delay when successfully implemented. This supports the interpretation that the longer total prehospital times observed in the video group are more likely related to transport distance than to delays introduced by the video intervention. Given the regional 45-minute triage threshold, patients eligible for video consultation were more often located farther from the thrombectomy center, whereas many patients in the non-video group were within this threshold and therefore transported directly without video. This selection mechanism likely contributed to systematically longer transport times in the video group, independent of the intervention. These findings underline that technical feasibility in individual consultations does not necessarily translate into routine feasibility in everyday practice.

A central finding was the perceived value of video consultation in supporting interprofessional collaboration. Both ANs and neurologists described how shared visual assessment enhanced mutual understanding, trust, and confidence in decision-making, particularly in cases with unclear or complex neurological presentations. This is consistent with previous studies showing that visual information can strengthen communication and reduce ambiguity in acute care [33–35]. These findings are particularly relevant when contrasted with the study by Bergrath et al., in which a tele-emergency physician with an anaesthesiology background supported paramedics using a structured checklist rather than direct specialist visual assessment [36]. While that model demonstrated organizational and technical feasibility, it relied on algorithm- or checklist-based decision-making rather than specialist-led neurological evaluation. The present study highlights an alternative telemedical configuration in which clinical responsibility remains clearly anchored with a stroke neurologist, and video consultation supports shared clinical reasoning rather than replacing local assessment. This distinction raises important questions regarding scope of practice, professional responsibility, and the relative reliability of checklist-based assessments versus specialist interpretation in prehospital stroke care. Neurologists in this study emphasized that ANs often perform structured and consistent neurological assessments, sometimes perceived as more reliable than assessments conducted by less experienced hospital physicians from other specialties. This observation aligns with Swedish and international research showing that prehospital stroke recognition can be highly accurate when supported by standardized scales such as NIHSS or mNIHSS [37–39]. Video consultation may further strengthen this process by allowing joint interpretation of findings, particularly in borderline cases. Previous work has shown that telephone-based pre-notification can shorten in-hospital treatment times and improve coordination of care [40]. However, telephone consultation primarily involves verbal information transfer. In contrast, video consultation allows remote specialists to directly observe neurological signs and engage in shared clinical reasoning with ambulance personnel.

Prior studies of prehospital video support for strokes mainly conducted in simulation or early feasibility settings suggest that real-time visual assessment may enhance diagnostic confidence and collaborative decision-making [24]. The present study extends this work by evaluating video consultation under real-world operational conditions.

In addition, video consultation may provide receiving hospitals with more detailed pre-arrival information, enabling better preparation and more efficient in-hospital workflows.

A contributing factor to observed redundancies in assessment was the rotation of multiple primaries on-call neurologists at the receiving hospital, who were not always fully familiar with all ongoing prehospital initiatives or research projects. Consequently, even when a video consultation had been performed, the receiving clinician could not always assume that this information was available or fully integrated into routine clinical workflows. This highlights that successful integration of prehospital video consultation requires not only technical solutions but also consistent awareness, shared protocols, and organizational alignment across all clinicians involved in the stroke pathway.

Although individual video consultations functioned well when successfully completed, overall feasibility was substantially limited by a high rate of unsuccessful or non-conducted consultations, primarily due to technical and organizational barriers. Feasibility should therefore be understood not only as technical functionality during successful use, but also in terms of reliability, accessibility, and frequency of use in routine practice. The combination of very few completed consultations (*n* = 5) and frequent technical failures makes it difficult to draw firm conclusions about feasibility in a broader sense. For this reason, the study should be regarded as exploratory. Its primary contribution lies in identifying barriers and enabling factors rather than demonstrating readiness for implementation. Future feasibility studies should ensure improved technical robustness, clearer organizational anchoring, and sufficient case volume to allow a more comprehensive assessment of feasibility under routine conditions.

The findings should also be interpreted in relation to major prehospital stroke triage trials, such as the RACE-CAT trial in Spain and the Stockholm Stroke Triage Study [41, 42]. These studies primarily evaluate algorithm-based triage using stroke severity scales to enable direct transport to regional thrombectomy centers and focus on optimizing large-scale, standardized pathways. In contrast, our study examined specialist-led video consultation as an extension of an existing telephone-based pathway, addressing a different implementation question: how real-time visual specialist support can function within routine ambulance care.

Rather than representing competing approaches, scale-based triage and video consultation may be complementary. Algorithms provide scalability, reproducibility, and system-level efficiency, whereas video consultation may add value in cases with atypical symptoms, uncertain scale results, or complex clinical presentations where structured scales alone may be insufficient. From this perspective, video consultation could function as an adjunct to established triage algorithms rather than a replacement, particularly in borderline or diagnostically challenging situations.

The very low number of completed video consultations (*n* = 5) should be interpreted considering the implementation context rather than as evidence against the concept of prehospital video support. During scale-up, responsibility gradually shifted from the project group to the regional organization, which reduced opportunities for hands-on training, feedback, and local troubleshooting. Combined with technical fragility (tablet availability, connectivity, and activation routines) and the inherently low frequency of eligible LVO cases per ambulance, this limited routinization and confidence among clinicians. These conditions suggest that successful use of video consultation depends not only on functional technology but also on sustained organizational ownership, continuous training, and clear activation pathways. A more detailed implementation-focused analysis of these barriers is addressed in a separate manuscript currently submitted for publication.

Prehospital telemedicine encompasses a wide range of approaches, including prenotification, digital documentation, decision-support systems, and video consultation [31, 40, 43]. However, the present study specifically focuses on video consultation. Compared with other telemedical approaches, video consultation is more resource intensive but also more interactive, allowing real-time dialogue and direct visual assessment. Implementation choices such as using fixed cameras in the ambulance cabin versus mobile camera devices, as well as factors like video stream stability and duration may influence usability and uptake. The present study illustrates that introducing video consultation is not merely a technical intervention but requires careful integration into existing workflows, equipment availability, training routines, and awareness across the entire stroke care chain. Without such integration, even clinically valuable technology risks remaining underutilized.

The qualitative findings highlighted that collaboration was generally perceived as respectful and equal, consistent with a previous Swedish study of video support in the prehospital stroke chain [44]. However, several barriers were identified, including technical unreliability, low frequency of use, and limited awareness among hospital staff. These findings reflect well-known challenges in telemedicine implementation and emphasize that successful adoption depends on both organizational and professional factors [30]. From an implementation perspective, clear role definitions, structured routines, and continuous training are essential to prevent uncertainty, particularly when ANs are required to assume a more active role during video consultations. Implementation frameworks such as Normalization Process Theory [45] may be useful in future studies to systematically analyse how video consultation can become embedded in routine practice. A more detailed implementation-focused analysis is addressed in a separate manuscript.

Despite these limitations, both ANs and neurologists perceived video consultation as clinically relevant and potentially timesaving, particularly by supporting direct transport decisions and avoiding unnecessary intermediate stops. Even modest reductions in time to reperfusion have been shown to result in meaningful improvements in functional outcomes after stroke [46, 47].

Future research should focus on larger, multicenter feasibility studies with improved technical reliability and clearer organizational integration. Such studies should not only assess technical performance but also examine determinants of use, interprofessional collaboration, and the role of video consultation within broader prehospital triage strategies. Only through such comprehensive evaluation can the true potential of prehospital video consultation in stroke care be determined.

### Limitations

This study has several important limitations. The most critical is the very small number of completed video consultations (*n* = 5), which prevents meaningful statistical comparisons and limits generalizability. Geographical and organizational differences between districts may have influenced process times, and the qualitative data reflected the experiences of only a subset of participants. Finally, inconsistent access to tablets and limited awareness among other hospital staff reduced the number of successful consultations, affecting overall feasibility evaluation. These factors should be considered when interpreting both quantitative and qualitative findings.

### Conclusion

This study demonstrates that prehospital video consultation is technically feasible in selected individual cases, but that substantial technical and organizational barriers limited routine use. While clinicians perceived the system as clinically useful, the very low number of completed consultations highlights the need for improved technical reliability and clearer organizational integration before broader implementation can be considered. Larger, multicenter feasibility studies are required to further evaluate the role of video consultation in prehospital stroke care.

## Data Availability

At this point the data is not available for further questions or consideration, please contact the corresponding author.

## Abbreviations

AN: Ambulance nurse
CT: Computed Tomography
EMS: Emergency Medical Services
LVO: Large Vessel Occlusion
mNIHSS: Modified National Institutes of Health Stroke Scale
NIHSS: National Institutes of Health Stroke Scale
SOS Alarm: Swedish dispatch center
ViPHS: Video consultation in the Prehospital Stroke Chain of care
